# Miniaturized Mass-Spectrometry-Based Analysis System for Fully Automated Examination of Conditioned Cell Culture Media

**DOI:** 10.1155/2012/290457

**Published:** 2012-10-04

**Authors:** Emanuel Weber, Martijn W. H. Pinkse, Eda Bener-Aksam, Michael J. Vellekoop, Peter D. E. M. Verhaert

**Affiliations:** ^1^Department of Biotechnology, Netherlands Proteomics Centre, Delft University of Technology, Julianalaan 67, 2628BC Delft, The Netherlands; ^2^Institute of Sensor and Actuator Systems, Vienna University of Technology, Gusshausstrasse 27-29/E366, 1040 Vienna, Austria; ^3^Biomedical Research Institute (BJOMED), Hasselt University, Agoralaan building C, 3590 Diepenbeek, Belgium

## Abstract

We present a fully automated setup for performing in-line mass spectrometry (MS) analysis of conditioned media in cell cultures, in particular focusing on the peptides therein. The goal is to assess peptides secreted by cells in different culture conditions. The developed system is compatible with MS as analytical technique, as this is one of the most powerful analysis methods for peptide detection and identification. Proof of concept was achieved using the well-known mating-factor signaling in baker's yeast, *Saccharomyces cerevisiae*. Our concept system holds 1 mL of cell culture medium and allows maintaining a yeast culture for, at least, 40 hours with continuous supernatant extraction (and medium replenishing). The device's small dimensions result in reduced costs for reagents and open perspectives towards full integration on-chip. Experimental data that can be obtained are time-resolved peptide profiles in a yeast culture, including information about the appearance of mating-factor-related peptides. We emphasize that the system operates without any manual intervention or pipetting steps, which allows for an improved overall sensitivity compared to non-automated alternatives. MS data confirmed previously reported aspects of the physiology of the yeast-mating process. Moreover, matingfactor breakdown products (as well as evidence for a potentially responsible protease) were found.

## 1. Introduction

In the field of proteomics/peptidomics mass spectrometry has become a well-established tool for protein/peptide sequencing [1–3]. Its steadily increasing performance (sensitivity as well as resolution) enables the analysis of thousands of different molecules at the same time which is of big advantage for “shotgun” approaches, where complex mixtures of unknown samples are targeted for identification. In combination with sophisticated separation methods, protein/peptide analysis has become much faster and more efficient [4–6].

Nevertheless, the whole analysis cycle, starting with peptide extraction from the medium of interest, sample pretreatments (chromatographic purification, digestion) prior to the ultimate injection in the MS instrument requires many time consuming and tedious steps, often done manually. Furthermore, the need for pipetting induces unavoidable sample losses, resulting in a decrease of the overall method sensitivity.

The goal of this work was the design and realization of a system, capable of performing sample extraction, protein/peptide enrichment, purification, and sample preparation for MALDI MS analysis in a fully automated and controlled manner. With the elimination of all previously necessary sample handling steps requiring pipetting, the sensitivity achievable by the system is boosted. Furthermore, using MALDI MS instead of direct connection to an ESI instrument allows for decoupling of the cell cultivation and the actual sample analysis. In that way those two parts can be performed independently from each other, even at different locations.

In addition, sample volumes are kept at a minimum. Reasons to pursue miniaturization include reagent costs. In many studies, different chemicals or additives are needed at certain concentrations to reveal activities of different components in the cell culture. The investment for additives is evidently reduced if the total sample volume is small. Besides these “economy” factors, evolution towards microscale is an essential step to a possible future design of a fully integrated, on-chip analysis system [7]. Once integrated on a single chip, all the advantages of those can be exploited, including (but not limited to) faster analysis cycles, implementation of extrasensing elements (e.g., viability analysis [8]) and on-chip temperature control [9, 10].

As a possible application of this system the analysis of cell-to-cell communication in *Saccharomyces cerevisiae* (baker's yeast) cultures based on peptide secretion was investigated. It is long known that peptides play an important role in cell-to-cell communication in yeast cultures [11]. As best documented example, we selected the mating process as model to evaluate the performance of our novel system. During mating, two yeast cells of opposite haplotype secrete a 13 amino acid pheromone called alpha-mating factor (secreted by alpha-type cells) and a 12 amino acid residue a-mating factor (released by a-haplotypes), respectively. This initiates alpha- and a-haplotype cell fusion to form a diploid cell [12]. In the course of this study the focus was on the detection, accumulation, and analysis of this peptide at different stages during cell culture growth. Therefore, cells were cultivated at small scale (1 mL) while continuously extracting and analyzing the extracellular conditioned medium. As a result a chronological sequence of MS spectra was obtained that could be nicely correlated to the corresponding growth stages. In a second study the effects of an enzyme inhibitor on potential peptidase activity cleaving alpha-mating factor was investigated. This experiment enabled us to collect evidence supporting the hypothesis for the involvement of a yapsin-like protease in an easy and fast way [13].

## 2. Materials and Methods

### 2.1. Strains and Growth Conditions

A WT *Saccharomyces cerevisiae* strain (CEN.PK 113-1A) mating-type alpha was used [14]. Cells were grown in mineral medium (MM) with addition of glucose (2%, w/v) as sole carbon source [15]. Prior to the transfer into the actual analysis chamber, yeast cells were precultivated in shaker flasks (10 mL MM, 30°C, 200 rpm). Such 24-hour culture has a typical optical density of 19-20 at a measuring wavelength of 600 nm (OD_600_). Dilution to an OD_600_ of 0.1 yielded the initial cell density chosen for all experiments. For every analysis, 1 mL of initial culture was transferred into a custom modified 2 mL glass vial which was prepared for connection to the analysis system.

In the protease inhibition experiments, 10 *μ*M pepstatin (Sigma Aldrich) was added to the culture flask after precultivation.

### 2.2. Miniaturized Cell Culture Chamber

**Figure 1:**
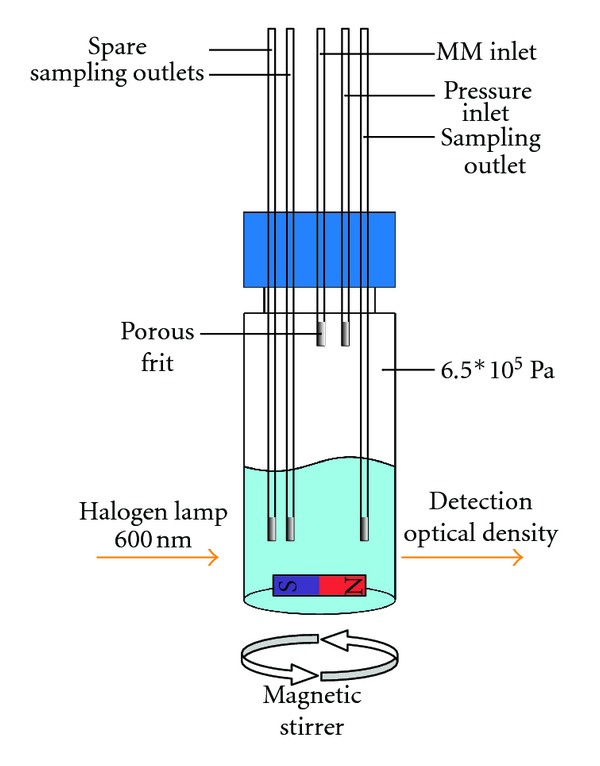
Schematic of miniaturized cell culture chamber: a modified 2 mL glass vial. Five fused silica tubings inserted through perforated cap provide liquid in- and outlets. Endings of fused silica tubings are fitted with a porous frit to prevent cells from leaving the vial and contaminating the analysis system. Overpressure of approximately 6.5 ∗ 10^5^ Pa inside chamber operates as pumping system. Efficient mixing of culture is ensured by minimagnet at vial bottom in combination with underlying magnetic stirrer. Light path for OD determination goes straight through vial between magnet and endings of fused silica.

A modified 2 mL glass vial with cap including septum (Agilent Technologies, USA) was used as a basic module for the miniaturized cell culture chamber. Fused silica tubings (inner diameter 100 *μ*m; BGB Analytik AG, Switzerland) were inserted through the perforated septum, to provide two liquid in- and three liquid outlets (Figure 1). All tubings inside the vial were fitted with a porous glass ending to allow cell culture medium to pass through, while preventing cells to leave the vial and enter the analysis system. Airtight closure of the vial, essential for the functionality of the system, was achieved by deposition of a silicone rubber-based sealant (Bison, Netherlands) on top of the cap. To obtain efficient mixing of the cell suspension the cell culture chamber was equipped with a small magnet and kept on a magnetic stirrer (500 rpm).

**Figure 2:**
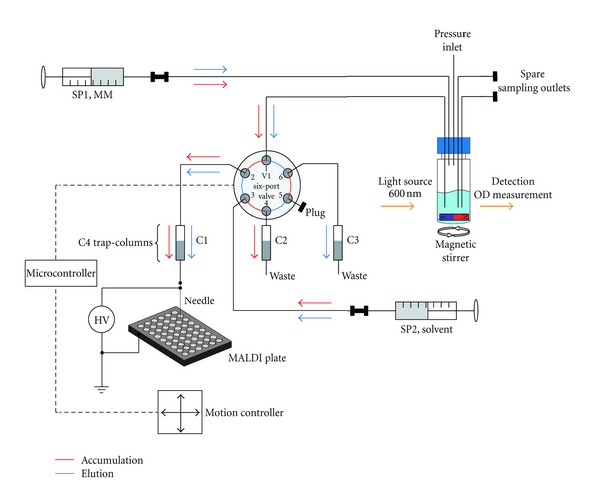
Overall analysis setup incorporating two syringes for MM and solvent supply and a switching valve for alternating between concentration/accumulation and elution steps. Cell culture chamber, placed on a magnetic stirrer, is connected via six-port valve with one of three C4 columns, which maintain a stable system backpressure. Via an electrospray needle held at 1.2 kV, sample is deposited onto a MALDI target plate.

Sampling of supernatant from the culture was done by creating an overpressure inside the vial. One of two inlets (“pressure”, Figure 1) was connected to a pressurized air system. The second inlet (“MM”, Figure 1) was connected to the medium reservoir (via syringe pump 1, SP1, Figure 2) for a constant supply of fresh mineral medium (MM, 0.5 *μ*L/min). In operation only one of the three outlets (“sampling”, Figure 1) was opened at a time, with the overpressure inside the chamber resulting in a steady sampling of supernatant.

### 2.3. Automated Setup for In-Line Sampling of Extracellular Medium System Components

The complete system consists of the cell culture chamber placed on a magnetic stirrer (IKA Labortechnik, Germany), two syringe pumps (Fusion 200; Chemyx, USA), a six-port valve (Rheodyne, USA) controlled by an external interface, three capillary columns packed with 5 *μ*m silica-based C4 beads (300 Å pore size, ReproSil; Dr. Maisch GmbH, Germany) for peptide enrichment/concentration and pressure stabilization, an electrospray unit for sample deposition, a MALDI target plate, and an *x-y-z* motion controller (MM4005; Newport, USA; Figure 2). The syringe pumps supply solvent for elution (SP2) and MM (SP1), keeping the volume in the vial constant. In the current experiments, no additional glucose or vitamins were supplied via SP1. The MALDI plate was accurately micropositioned by the motion controller. An in-house software program was developed and loaded into the microcontroller of the MM4005 for synchronization with the six-port valve. Real-time determination of optical density was realized with a fiber spectrophotometer (Avaspec-2048) and suitable light source (DH-2000; Avantes, Netherlands). MS analysis was performed directly from the spotted samples in a MALDI Q-TOF mass spectrometer (QTof Premier; Waters, Manchester, UK), equipped with a solid state NdYag laser.

### 2.4. Real-Time Optical Density Measurement inside the Miniaturized Cell Culture Chamber

**Figure 3:**
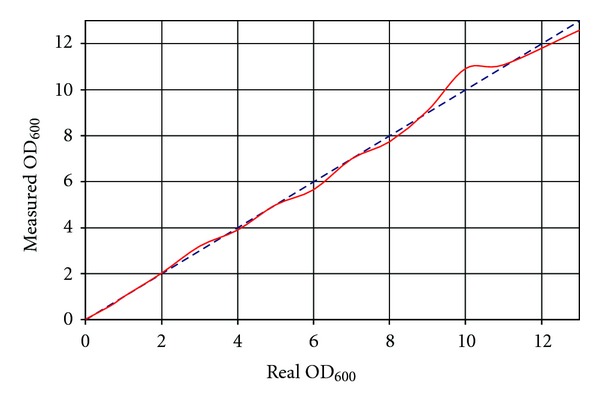
Calibration curve for linearization of OD_600_ measurement. Blue dashed line represents optimal behavior. Solid red curve illustrates actually determined OD_600_ after measuring, averaging, and calibration.

The optical density was measured with a fiber spectrophotometer setup especially conceived for use with 2 mL glass vials. Initial linearity of the device at 600 nm was established for ODs between 0.1 up to 1.5. An extended calibration curve was recorded to get valid data at higher densities as well. After averaging of more than 200 individual measurements for one data point and curve fitting (Matlab, The MathWorks, USA) linearity was obtained for values up to OD_600_ 13, fully covering the range of interest (Figure 3).

### 2.5. Sampling Cycle Operation

Operation of the sampling system was basically divided into two parts: (i) sample accumulation/concentration and (ii) elution. The temporal resolution of the setup in the current configuration is approximately 2 hours. Keeping in mind the life cycle of *S. cerevisiae* (reproduction/cell division each 75–120 minutes [16]) this resolution gives chronological information about the state the whole culture is going through rather than information at the single-cell level. In the accumulation step (90 min) the analysis column C1 was connected directly with the open outlet of the cell culture chamber via valve V1 (Figure 2). The outflow of solution in that time was spotted onto a waste position on the MALDI plate. The flow was adjusted to 0.4–0.6 *μ*L/min resulting in a sampling volume of 36 to 54 *μ*L over the 90 minutes accumulation/concentration period. During this step solvent was pumped through column C2 (Figure 2) connected via the valve to waste. The inclusion of column C2 proved necessary for maintaining constant backpressure inside the system and hence constant (out-) flow. After accumulation, elution followed by switching the valve, which simultaneously triggered the motion controller to position the first spot on the MALDI plate exactly under the electrospray unit. Solvent (water/acetonitrile/acetic acid; 10 : 90 : 0.6, v/v/v) was pumped through the column C1 at a flow rate of 1 *μ*L/min. Eluates were deposited for 1.5 min per spot (corresponding to 1.5 *μ*L). Ten sample spots were collected in a row to ensure complete elution of the column. Carryover between consecutive analysis runs can be excluded as empty MS spectra (no peptide ion peaks) were obtained for the last sample spots of each series. Extraction of supernatant out of the cell culture chamber continued during the elution step as well. The sample was continuously pushed through a second waste column (C3, Figure 2) for flow stabilization reasons. Both, accumulation and elution step, were repeated up to 11 times, equivalent to more than 19 hours of total analysis time. Throughout the experiment the optical density was measured at a 2 hours interval, and the resulting growth curve was recorded (see e.g., Figure 5).

### 2.6. Preparation of Target Plate for MALDI Mass Spectrometry

Prior to sample spotting, the MALDI plate was ultrasonically cleaned in ammonium bicarbonate solution (10 mM) followed by water/methanol/trifluoroacetic acid (50/50/0.1, v/v/v). Alpha-cyano-4-hydroxycinnamic acid was used as matrix (dissolved at 6 mg/mL in water/acetonitrile/trifluoroacetic acid; 50/50/0.1, v/v/v). After electrospray deposition of the samples, 0.8 *μ*L of matrix solution was added to each spot on the MALDI plate. The plate was analyzed in the MS system, i.c. MALDI Q-TOF after air-drying and complete crystallization of the matrix.

Direct connection of the presented setup to an ESI MS instrument is feasible but requires both parts, cell cultivation as well as sample analysis, to be physically linked which prohibits independent operation and requires all instruments to be placed at the same location.

## 3. Results

### 3.1. Detection of *S. cerevisiae* Mating Factor

**Figure 4:**
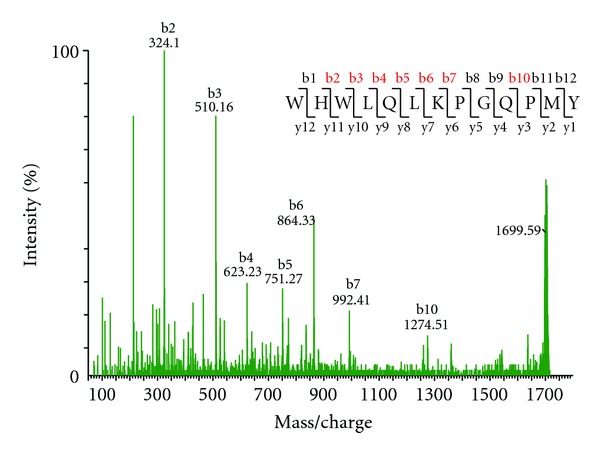
MS/MS spectrum of alpha-mating factor (oxidized at Met_12_). Insert: amino-acid sequence with detected sequence ions indicated in red. Note virtually complete b-ions series.

Alpha-pheromone (TrpHisTrpLeuGlnLeuLysProGlyGlnProMetTyr, monoisotopic mass 1682.84 Da) is detected in our MALDI Q-TOF MS as [M + H]^+^ at *m/z* 1683.85. Associated with this ion often a peak at *m/z* 1699.84 is observed, corresponding to the peptide oxidized at position Met_12_ (a very common posttranslational modification (PTM)). The identity of the peptide could be confirmed by CID of the 1699.84 precursor ion (MS/MS spectrum given in Figure 4).

The alpha-mating-factor-related peptides were detected nearly throughout the whole analysis indicating that alpha factor is expressed and secreted constitutively (also in the absence of opposing-mating-type cells/pheromone). The appearance of alpha-mating factor was more obvious at the late exponential growth phase (corresponding to the diauxic shift, when conditions get less favorable, after nutrient consumption). Two major fragments of the alpha-mating factor (the aminoterminal hexapeptide and the carboxyterminal heptapeptide) were detected in the medium (verified by MS/MS, data not shown) besides the intact pheromone. These two mating-factor (degradation-) fragments may be products of a protease which cleaves the intact pheromone in two pieces (see Section 3.4 [17]). The fact that their amounts increase in time while intact pheromone decreases at later stages of growth would agree with this.

### 3.2. Real-Time Monitoring of *S. cerevisiae* Growth in Cell Culture Chamber

**Figure 5:**
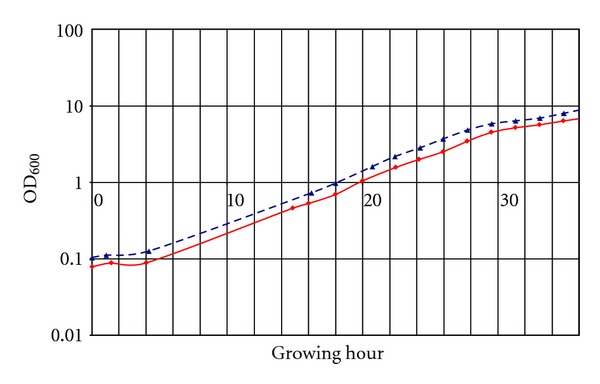
Growth curves recorded from two cultures grown under different conditions (blue, dashed curve represents standard growing conditions; red, solid curve with addition of protease inhibitor pepstatin). A small time lag between both curves is evident and makes a comparison based on absolute time points inaccurate.

**Figure 6:**
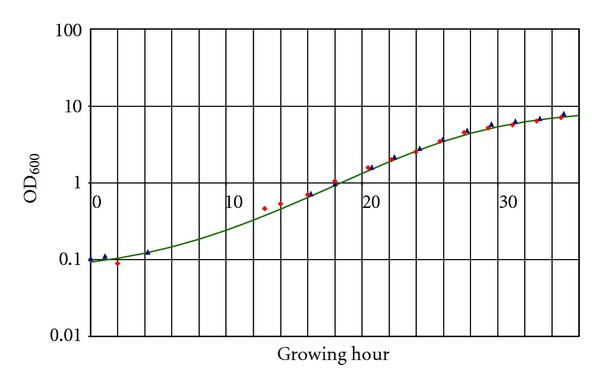
After PC-supported adjustment of measurement series of pepstatin-containing culture a perfect matching of both growth curves was obtained. All points lie on fitted sigmoid function (solid, green curve), allowing a valid comparison.

The objective of this study was to develop an automated setup for the analysis of conditioned media of *Saccharomyces cerevisiae* cultures at different growth stages at a miniature scale. To obtain reproducible results and to allow valid comparisons between experiments, it is important to keep the cells at the same physiological/growth state for all experiments. The growth state of the cells was monitored robustly by measuring cell density. For this a fiber spectrophotometer was integrated in our setup, specifically designed for use with 2 mL glass vials. This allowed real-time noninvasive determination of culture ODs. To cover the whole range of ODs a typical yeast culture under the applied conditions goes through (0.1 to 13), a thoroughly elaborated calibration including multiple individual measurements was generated. The real-time recorded growth curves were accurate, as they were in excellent accordance with calibration curves obtained from standard OD determination techniques (diluting the culture to ODs in the linear range of the spectrophotometer and recalculating the actual OD, data not shown). Our analyses confirmed that different batches of yeast cultures show very similar growth behaviors (Figure 5). However, for a meaningful comparison between cultures at various time points/growth stages a perfect match of the two curves is essential. Parameters like small variations in the initial ODs of the inoculated culture or the addition of a test compound may result in slightly delayed or shifted initiation of cell growth. This can be corrected for by software-wise adjustment (“warping”) of one of the two curves onto the other (a simple shift along the time axis often being sufficient). The finally obtained diagram shows a perfect match in terms of cell growth of both investigated conditions (Figure 6). All timepoints of both series overlap on a single curve which facilitates a valid comparison.

### 3.3. Time-Resolved Detection of Mating Factor (and Other Peptide-Like Compounds) in Yeast Cell Culture Media under Standard Growth Conditions

To study the cell culture medium during a standard yeast growth, samples were collected at 2 hours intervals starting at an OD_600_ of approx. 1, which typically is reached 18 to 20 hours after inoculation of the initial culture (OD_600_ of 0.1).

**Figure 7:**
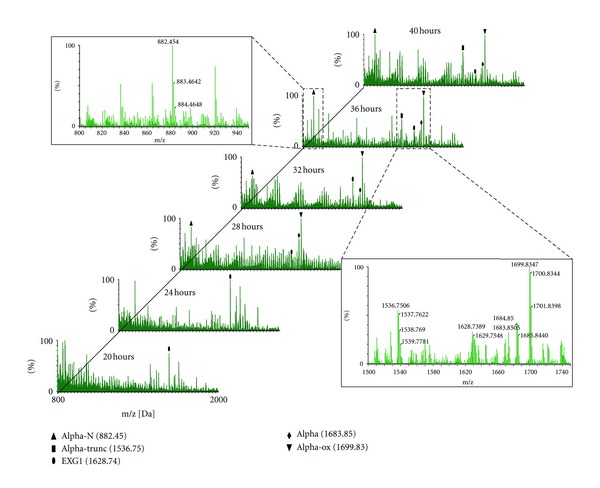
MS spectra at 2 hours intervals during standard yeast growth (for figure clarity, every 2nd spectrum is omitted resulting in a 4 hours interval). Marked peaks (legend insert) indicate peptide ions of interest (alpha-N, 882.45 Da: [M + H]^+^ of aminoterminal mating factor hexapeptide; alpha-trunc, 1536.75 Da: carboxy terminally truncated oxidized mating factor (oxidized alpha factor minus C-terminal tyrosine residue); EXG1, 1628.74 Da: fragment of EXG1 membrane protein; alpha, 1683.85 Da and alpha-ox, 1699.83 Da: alpha-mating factor and its oxidized version). Mating-factor-related peptides do not appear at early time points, whereas they become the most abundant ion peaks at later growth stages, which fits with physiological data. Inserts (top left; bottom right) show magnifications of a spectrum with annotated peaks of interest and their isotopes.

Up to 11 consecutive samples were analyzed by MALDI Q-TOF MS(/MS) yielding chronologically classified MS spectra (“peptide profiles”).  Figure 7 shows representative spectra at every second time point sampled (resulting in a difference of 4 hours between each consecutive spectrum displayed). Several different peptide-like signals are evident in the mass spectra acquired. To assist in the interpretation of these profiles, 5 mass over charge (*m/z*) values of interest are highlighted throughout all the spectra, (four between 1520 and 1720 *m/z* and one at about half the *m/z*). MS/MS analysis confirmed that the peaks at *m/z* 1683.85, 1699.83, and 1536.75 represent the mating factor, its oxidized version, and a C terminally truncated species (loss of Tyr residue), respectively. The peak in the lower *m/z* region, at 882.45, represents one of the two mating-factor cleavage products (the aminoterminal hexapeptide). The complementary peptide fragment (the carboxyterminal heptapeptide) was not readily identified.

One of the most distinct peptide peaks shows up at 1628.74 Da (Figure 7). MS/MS analysis and Mascot database searching (using “no enzyme” as parameter) identified it as the soluble fragment of a cell wall protein; exo-1,3-beta-glucanase (EXG1). EXG1 is known to be involved in cell wall organization by enabling beta-glucan assembly [18]. Literature data [19] and our time-course analysis confirm its presence in the culture medium just before alpha-mating-factor secretion. This suggests that this protein fragment is shed from the membrane just prior to, or simultaneously with, mating-factor release, which may imply a potential role of this protein fragment in mating.

For the time-resolved detection of mating-factor, cells were grown in MM without addition of any special component except those needed for cell growth. It was observed that none of the four mating-factor-related peaks appear early during growth. First unequivocal detection is between hour 26 and 28, that is, at an OD_600_ of approx. 3.5. Mating-factor concentrations significantly increase in the later growth stages. It is remarkable that both intact mating factor and one of its fragments (the aminoterminal hexapeptide) are detected virtually simultaneously. Multiple repetitions (exceeding 5) confirmed the above-stated behavior.  Figure 7 should be understood as an illustrative sequence of MS spectra of a single continuous (40 hours) analysis run.

### 3.4. Effect of Pepstatin (Inhibition of Aspartic Proteases)

**Figure 8:**
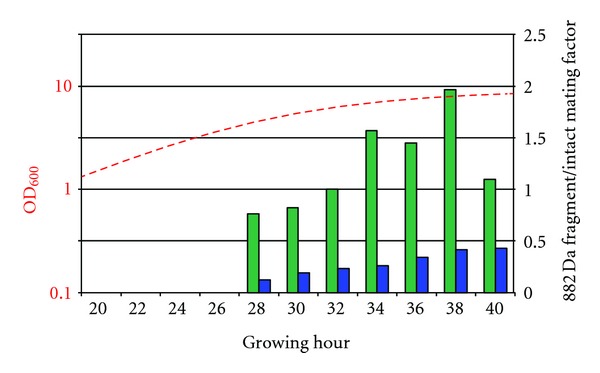
Ratio of 881.45 Da fragment to intact mating factor (1682.84 Da plus 1698.83 Da; right scale). No mating factor or fragments are detected in first 4 investigated time points. At later time points ratios between 0.8 and 2 for culture grown under standard conditions were obtained (right scale, green bars). Note significant reduction of relative amount of 881.45 Da fragments in pepstatin-containing culture (blue bars). Dashed, red curve (left, logarithmic scale) plots growth curve of cells.

To illustrate the usefulness of the miniature culture media analysis system, a simple experiment was designed looking at the effect of a protease inhibitor on the appearance of selected peptide fragments detected above. The proteolytic cleavage of mating factor at the Leu/Lys peptide bond suggests involvement of an aspartic protease. Hence a general aspartic protease inhibitor was selected to study its effect on the appearance of the peptides/peptide fragments observed [20]. Pepstatin was added to the culture at a concentration of 10 *μ*M. The anticipated effect reduced appearance of the 881.45 Da fragment ([M + H]^+^ at *m/z* 882.45) during the whole experiment. Besides the addition of 10 *μ*M of pepstatin all conditions were kept strictly the same as for the other experiments. The first appearance of intact mating factor and its oxidized variant was observed at the same time point as for the culture without inhibitor. This indicates that the addition of pepstatin at the chosen concentration had no effect on the actual secretion of alpha factor (MS data not shown). The abundance of the 881.45 Da fragment on the other hand was significantly lower in all spectra (Figure 8, right scale). The red, dashed line in Figure 8 (left scale) depicts the growth curve (in terms of optical density (light absorbance) at 600 nm) at the time of sampling. Data shown represent values from a complete analysis run lasting over 40 hours. Values plotted are representative as biological repetitions of the experiment over shorter time frames showed the very same trend. 

## 4. Discussion

### 4.1. Alpha-Mating-Factor Profiles at Different Growth Stages

During the yeast life cycle, mating factor is the trigger for two haploid cells of opposite mating type (alpha and a) to mate and form one single diploid cell. This happens in nature once the growth conditions get unfavorable, for example, lack of nutrition. The strain used in this study is incapable of changing its sex/haplotype [14]. This precludes the formation of diploid cells in the culture flask. However, it is clear that these haploid cells still produce their pheromone in the absence of an opposing mating type or pheromone. The obtained MS spectra in Figure 7 illustrate that the cells “signal for mating” particularly at the later stages of growth, that is, at the end of and after the exponential growth phase. In the early stages of growth no peaks representing the mating factor were identified.

### 4.2. Alpha-Mating-Factor Detection in Pepstatin-Containing Cultures

Comparing the growth curves of the pepstatin containing with those of “standard” cultures confirmed that the growth behavior of the cells was similar in both conditions, justifying a valid time-based peptide profile comparison (Figure 6).

We hypothesized that if mating factor is secreted already at an earlier cell growth stage but readily cleaved by a protease, inhibition or inactivation of this protease could promote the detection of intact mating factor at an earlier time point in the growth curve. This was not observed. The intact mating factor appeared at the same time in both experiments. However, it should be noted that the time resolution of the current setup was 2 hours. Small shifts within this interval may still have been missed.

### 4.3. Effect of Pepstatin on Mating-Factor Fragment Appearance

The comparison of cell cultures with and without pepstatin showed significant differences in terms of the extracellular peptide profiles. The presumed aspartic protease responsible for the formation of the 881.45 Da fragment clearly seems to be inhibited by pepstatin. At all examined time points the ratio between the overall count of the 881.45 Da fragment and that of the intact mating factor (both native (1682.84 Da) and oxidized (1698.83 Da)) was significantly decreased in the culture containing the protease inhibitor (Figure 8). Only at the latest points of inspection, the stationary stage of cell growth, a noticeable count of the 881.45 Da fragment was detected but still far below the intensity level of that in the pepstatin-free culture. Given that the conditions for both cultures were kept identical, the disappearance or drastic reduction of the fragment in the extracellular medium is clearly to be attributed primarily to the addition of pepstatin. This suggests that pepstatin inhibits the potential protease responsible for “normal” alpha-mating-factor peptide cutting.

### 4.4. Additional Mating-Factor-Related Peptide Ions

In the obtained MS spectra additional mating-factor-related ions were identified. The detection of oxidized mating factor missing the tyrosine at the C-terminus at the later growth stages (*m/z* 1536.75; Figure 7) may reflect the action of an (carboxyterminal) exopeptidase in the extracellular medium. Concurrently, the amount of intact mating factor inside the culture decreases.

### 4.5. Other Nonmating Factor-Related Peptide Ions

Besides ions related to mating factor, another peptide possibly involved in the secretion process was identified (*m/z* 1628.74; Figure 7). Database searching identified it as the soluble part of the exo-b-1,3-glucanase EXG1 (a cell wall protein). The role of this protein in peptide secretion remains elusive, but comparing its appearance in both experiments showed marked differences. In standard cultures, the peptide was found most abundant prior to secretion of mating factor and slightly reduced at later growth stage. In-pepstatin containing cultures this peptide was not found prior to mating peptide secretion, and it appeared considerably less prominently present at later growth stages as well (data not shown).

## 5. Conclusion

The presented automated system allows in-line sampling of microliter amounts of extracellular conditioned cell culture media, preparing them for MALDI MS analysis. The minimal amount of cell culture required for this has advantages in terms of handling and cost reduction. For example, compared to standard flask cultivation, an enzyme inhibition study during cell growth could be completed with 10 times less amount of the commercial compound (i.c. pepstatin). In particular when effects on cultured cells of more expensive compounds have to be tested, the experiment cost savings related to the reduced culture chamber volume will become more substantial.

Implementation of the real-time optical density measurement in-line (without disturbing the cell culture) made many tedious extra sample collection and dilution steps redundant and resulted in an overall increase of the practicability of the system.

In summary, we have realized a fully automated setup which eliminates all manual pipetting interventions. This reduces the risk for losses of peptides sticking to microtip or tubing/column wall materials, which often drastically reduces the overall sensitivity of the analysis.
